# Outcome of unilateral lateral rectus recession and medial rectus resection in primary exotropia

**DOI:** 10.1186/1756-0500-6-257

**Published:** 2013-07-08

**Authors:** Quratul Ain Saleem, Alyscia M Cheema, Muhammad Ali Tahir, Arif Rabbani Dahri, Tahir M Sabir, Javed H Niazi

**Affiliations:** 1 Department of Ophthalmology, JPMC, Karachi, Pakistan

**Keywords:** Unilateral recession, Resection, Primary exotropia, Prism Diopter (PD)

## Abstract

**Background:**

The purpose of this study was to measure the success rate of unilateral lateral rectus recession and medial rectus resection in primary exotropia.

**Methods:**

This is an interventional case series of 55 patients with primary exotropia (degree of deviation 15–85 PD), above the age of 5 years. Patients were treated in the Department of Ophthalmology, Jinnah Postgraduate Medical Center, Karachi, Pakistan, during the period of July 2009 to March 2010. All the patients underwent surgical procedure i.e., lateral rectus muscle recession (maximum up to 10 mm) and medial rectus muscle resection (up to 6 mm) of one eye, according to the Park’s method. Surgery was done based on prism cover test measurements obtained at 6 m with appropriate optical correction in place. Patients were re evaluated at one day, one month, two months and six months post operatively. Final outcome was considered at the end of six months at which achievement of ≤10 PD of exotropia was the success. Data was analyzed on SPSS version 17.0.

**Results:**

We obtained success (≤10 PD) in 42 out of 55 patients (76.4%) and 13 out of 55 patients (23.6%) did not meet our criteria for surgical success (>10 PD). Analysis of success with the type of primary exotropia showed that success was achieved in 22 out of 24 cases of intermittent type (91.6%) and 20 out of 31 cases of constant type (64.5%)(P Value 0.019). The highest percentage of success was achieved in patients with the pre-operative deviation of ≤70 PD i.e., 93.3% (42 out of 45 cases), while none of the patients with the pre-operative deviation of >70 PD (10 out of 10 cases) achieved the criteria for success.

**Conclusion:**

We conclude that pre-operative deviation is one of the strongest predictor for favorable surgical outcome. Therefore, eliminating the factors causing error in the correct determination of pre-operative deviation should improve the success and predictability of the surgical outcome. Despite the obstacles in the surgical management of strabismus, our results are encouraging.

## Background

Exotropia is a disorder of ocular alignment characterized by an outward deviation of the eyes. It is a common condition affecting approximately 1% of all children under 11 years of age [1]. Previous Western studies have traditionally suggested that convergent strabismus (esotropia) was twice as common as divergent strabismus (exotropia). Recent studies, however, suggest that the reverse may be true in Asian population. Exotropia is classified as Primary which includes constant and intermittent exotropias. Among exotropia the intermittent exotropia is the commonest type [2]. Sensory and consecutive exotropias are called secondary because deviation is secondary to media opacity or fundus pathology and resulting from overcorrection of esotropia respectively [3]. Constant exotropia is characterized by a constant angle of deviation. In intermittent exotropia eyes are straight with binocular vision at times and manifest with suppression on deviation at other times [4]. Intermittent exotropia has been further classified by Duane’s classification as the divergence excess type of exotropia, convergence insufficiency type of exotropia. and the basic type of exotropia [5].

Untreated poorly controlled intermittent exotropia later progresses to constant exotropia [6]. Poor control suggests that deviation is present most of the time, easy to break down and do not recover well after occlusion [7]. Treatment is indicated to restore binocular function and to reconstruct normal ocular alignment [8]. Surgery is the preferred treatment for symptomatic (manifest) exotropias [9]. Non-surgical treatment may be indicated to optimize sensory condition before surgery [8].

Traditional surgical treatment has been two muscle surgery i,e. unilateral lateral rectus recession with medial rectus resection and bilateral lateral rectus recession, although unilateral lateral rectus recession is an alternative for small angle exotropias [10]. The choice of procedure classically has been based on the measured distance/near deviation. Parks has shown that bilateral lateral rectus recessions work well for all three types of intermittent exotropias [11]. Kushner has suggested that patients with basic type intermittent exotropia should be treated with unilateral recess/resect procedure [12]. Unilateral lateral rectus recession and medial rectus resection procedure has the success rate of 83.3% and that of bilateral lateral rectus recession is 48.3% in international studies [13]. Unfortunately no local data is available on this topic. The rationale of my study is to assess whether ocular alignment can be successfully achieved with two muscle surgery i.e., by unilateral lateral rectus recession and medial rectus resection, or may require third or fourth muscle to be operated upon.

This study will be helpful in developing and recommending a standard procedure in order to achieve ocular alignment in patients with primary exotropia. It is a cosmetic surgery that does not affect the vision, anterior segment findings, fundus findings and retinoscopy.

## Method

This is an interventional case series of 55 patients with primary exotropia (degree of deviation 15–85 PD) of either sex and above the age of 5 years. It was carried out at the department of Ophthalmology (ward-11) of Jinnah Postgraduate.

Medical Center, Karachi, Pakistan, during the period of July 2009 to March 2010. Patients with oblique muscle dysfunction, vertical strabismus, A-V pattern strabismus, past history of extra-ocular muscle surgery, paralytic strabismus and other ocular disease e.g., congenital cataract, retinal detachment etc. were excluded from this study. All patients underwent an ophthalmological examination before surgery. A visual acuity test was performed with the correction of refractive errors. Refractive error was measured after topical administration of 1% cyclopentolate in younger patients, and by a manifest refraction in adults. For patients with myopia, full cycloplegic refraction was dispensed. For patients with hyperopia, spectacles were prescribed if there was any Substantial astigmatic refractive error, anisometropia >1.50 diopters, or hyperopia >2.00 diopters. In most cases, patients with hyperopic intermittent exotropia were given spectacles that incorporated approximately 1.50 diopters less than the full cycloplegic hyperopic refraction. Patients with a difference of two lines or more of visual acuity in each eye were considered to have amblyopia and underwent part-time or full-time occlusion along with spectacle correction before operation until no further improvement was observed after 3 consecutive visits with a three-month interval. Sensory testing was performed using the Worth-4-dot, Titmus fly, and Lang tests. The angle of deviation was determined by the prism and alternate cover technique with an accommodative target at both distance and near. The accommodative target fixation point was at 6 m (far) and 1/3 m (near). An additional prism and alternate cover test was performed for co-operative patients while they were looking through a window, fixated on a distant outdoor target.

Thus all patients diagnosed having primary exotropia i.e., constant and intermittent (poorly controlled and basic type) were selected for this study based on inclusion and exclusion criteria. The lower age for inclusion was 5 years as I wanted to limit my study to the patients in whom accurate measurements could be obtained with the prism cover test. The selection was completed after permission of the Jinnah Postgraduate Medical Centre Ethical Committee and informed consent which was taken from the patients and in case of small children from their parents. Purpose and procedure of the study were explained to all patients. After explaination of the surgical procedure patients underwent surgery i.e., lateral rectus muscle recession (maximum upto 10 mm) and medial rectus muscle resection (upto 6 mm) of one eye, according to the park’s method (Table 1) [14] based on prism cover test measurements obtained with the appropriate optical correction in place. Surgery was performed by a single surgeon under local anaesthesia in adults but children were operated under general anaesthesia.

**Table 1:** Surgical dosage for patients with primary exotropia

**Prism diopters**	**Recession amount of lateral rectus(mm)**	**Resection amount of medial rectus(mm)**
25	4	3
30	4	4
35	5	4
40	5	5
45	7	5
50	8	5
55	8	6
60	10	6

The angle of deviation was first evaluated on 1st post operative day. Serial follow up visits were scheduled at one month, two months and six months post operatively. Final outcome was considered at the end of six months at which achievement of ≤10 PD of exotropia was defined as success.

The data was filled in Performa and analyzed on SPSS version 17.0. The qualitative data such as gender and success were presented by their frequencies along with percentages. The continuous variables such as age and degree of exotropia in PD was presented as mean ±SD. Stratification was done with regards to age, gender, degree of exotropia (in PD) and the types of primary exotropia in order to see the impact of these variable on the outcome. Comparisons between types of exotropia and their success were performed using Pearson Chi-square. P -Value ≤0.05 was considered as significant.

## Results

Fifty five patients of primary exotropia met the inclusion criteria described in the methodology. There were 25 males (45.5%) and 30 were females (54.5%) (M:F 1:1.2). Their mean ages were 16.45±7.42 years (range 5 to 35 years). Unilateral amblyopia was present in 15 (27%) patients thus occlusion therapy was performed preoperatively in these patients, out of which 3 (20%) patients attained the equal visual acuity binocularly, 7 (47%) patients improved with full-time occlusion and spectacle correction but did not achieve the visual acuity of the fellow eye while 5 (33%) patients did not improve at all. Rest of the patients had 6/6 visual acuity in each eye. Twenty four patients (43.6%) had intermittent type of primary exotropia, whereas 31 patients (56.4%) had constant type of primary exotropia. The minimum pre-operative deviation seen was 40 PD and maximum was 85 PD (mean 59.45±12.862 PD). The minimum post-operative alignment achieved was 8 PD and maximum was 30 PD (mean 11.05 PD). We obtained success (≤10 PD) in 42 out of 55 patients (76.4%), while remaining 13 (23.6%) did not meet our criteria for surgical success (>10 PD), i.e., they were under corrected. However there was no case of overcorrection in our study population.

Analysis of success with the type of primary exotropia showed that success was achieved in 22 out of 24 cases of intermittent type (91.6%) and 20 out of 31 cases of constant type (64.5%) (P value= 0.019) (Table 2). Data analysis with respect to the pre-operative deviation showed that the highest percentage of success was achieved in patients with the pre-operative deviation of ≤70 PD i.e., 93.3% (42 out of 45 cases), while none of the patients with the pre-operative deviation of >70 PD (10 out of 10 cases) achieved the criteria for success. Thus the success rate in patients with the pre-operative deviation greater than 70 PD differs from those patients who had the pre-operative deviation less than 70 PD (Table 3).

**Table 2:** Effect of the types of primary exotropia on success

**Type**	**Success**		**Total**
	**Yes**	**No**	
**Intermittent**	**22**	**2**	**24**
**Constant**	**21**	**10**	**31**
**Total**	**43**	**12**	**55**

**Table 3:** **Effect of pre**-**operative deviations on success**

**Pre**-**operative deviation(PD)**	**Success**		**Total**
	**Yes**	**No**	
**40**	**6**	**0**	**6**
**45**	**3**	**0**	**3**
**50**	**10**	**0**	**10**
**55**	**8**	**0**	**8**
**60**	**8**	**0**	**8**
**65**	**4**	**0**	**4**
**70**	**3**	**3**	**6**
**75**	**0**	**4**	**4**
**80**	**0**	**3**	**3**
**85**	**0**	**3**	**3**
**Total**	**42**	**13**	**55**

## Discussion

Many patients with intermittent exotropia ultimately require surgery. Several surgical approaches have been used successfully to treat exotropia. The choice of procedure classically has been based on the measured distance/near differences. Kushner has suggested that the patients with basic type primary exotropia should be treated with unilateral recess/resect procedure [12]. However for the surgical treatment of large-angle exotropia binocular surgery is the most commonly used approach involving three or four horizontal rectus muscles. This procedure is widely used because it avoids significant limitations of ocular movement, which could occur in surgeries of greater magnitude. In earlier publications, [15,16] many ophthalmologists chose to operate on two horizontal muscles and correct residual deviations with second or third procedures with variable results. Others, who were in the minority, considered simultaneous four horizontal muscle surgery to be a superior approach for these deviations [17,18]. However, we choose to surgically correct primary exotropia with the unilateral recess/resect surgery because it has more advantages as it preserves some muscles if a repeat operation is required, avoiding the exposure of the dominant eye to the inherent risks of a surgical procedure and reducing surgical time.

In our study there was a slight female predominance similarly observed in Gezer study where female were (58.2%) and male patients (47.8%) [19]. On the contrary, Livir-Rallatos G reported the clinical records of 63 patients out of which 33 were males and 30 were females [20]. Thus different studies showed slight variation in the gender distribution.

Since females are more concerned about their cosmetic appearance this seems to be a reason for more female patients undergoing surgery in our study.

Ages of the patients in our study ranged from 5 to 35 years (mean age 16.45 years, standard deviation ±7.42 years). In a similar study conducted by Jeoung JW the average age of the patients at the time of the surgery was less i.e., 7.8 ±5.9 years (mean ± standard deviation, ranging from 2 to 53 years) [13]. However in a study conducted at Cameroon by Mvogo et.al, [21] the average age was 18.7 years ±11.2 that was closer to our study. The high average age at the time of operation in our study is because people hardly accept surgery in children, more so when it concerns the eyes. Moreover, the time lapse between the diagnosis and surgery is very important as it can aggravate the amplitude of the strabismus angle. In effect, patients who have witnessed operated cases are more motivated for surgery. However, according to Koo et al., [22] the age at operation of strabismus does not influence the postoperative result. The optimum age for surgery is when the child is able to undergo orthoptic assessment and/or when the functional and cosmetic symptoms become evident [23-25].

In our series, higher success was achieved in intermittent type of exotropia as compared to constant type of exotropia. Similar results were observed in Lau F H S in his study of 24 consecutive patients reported higher success rate in the intermittent group (88.2%) than in the constant group (42.9%) [26]. Similarly Richard, Parks and Stoller’s study has shown intermittent exotropias having the best operative outcome [25-27]. However Gezer in a study reported 89 patients (39.5%) having intermittent exotropia, and 136 patients (60.5%) having constant-type exotropia. In his analysis the type of exotropia was found to be insignificant in influencing the surgical outcome of exotropia [19]. Similarly Mvogo in his series of 43 patients, out of which 6 patients (14.7%) had intermittent and 35 patients (85.3%) had constant type of primary exotropia, did not find any differences in the surgical results with respect to type of primary exotropia [21].

In our study minimum pre-operative deviation seen was 40 PD and maximum seen was 85 PD (mean 59.45 PD). The minimum post-operative alignment achieved was 8 PD and maximum was 30 PD (mean 11.05 PD). Data analysis with respect to the pre-operative deviation showed that the highest percentage of success was achieved in patients with the pre-operative deviation of ≤70 PD i.e., 93.3% (42 out of 45 cases). All the patients with the pre-operative deviation of >70 PD (10 out of 10 cases) failed to achieve the criteria for success. Thus the success rate in patients with the pre-operative deviation greater than 70 PD differs from those patients who had the pre-operative deviation less than 70 PD.

The definition of a successful outcome depends entirely on the criteria used for such success. Many define satisfactory alignment as within 10 PD of orthotropia, [13,23,27] while others extend the criteria to within 15 PD [28,29].

In a local study conducted by Junejo S A, where success was defined as ≤15 PD, they were able to achieve the overall success rate of 73.3% in exotropia patients with the pre-operative deviations in the range of 30 to 60 PD. The pre-operative deviations of 60 PD or less illustrated success with the unilateral recess/resect procedure, but they stated difficulty in visually aligning the eyes by operating only on one eye because of larger deviations of more than 60 PD. Thus according to them at this stage, clinical circumstances for a second surgery on the fixing eye or binocular squint surgery concerning more than two horizontal rectus muscles is the main and extensive used clinical procedure [29]. Jeoung et.al, in his comparative study found the significantly higher surgical success in patients with the unilateral recess/resect procedure than those having bilateral lateral rectus recession. Thus his overall success in the recess/resect procedure group was 83.3%. They defined success as ≤10 PD. A higher success rate in this study comparative to us (i.e., 76.4%) is because of comparatively less pre-operative deviations i.e., from 14 to 45 PD (mean, 27.0 ±7.5 PD) in the patients that were included in the study, as pre-operative deviations in my study was from 40 to 85 PD (mean 59.45 PD) [13]. Millan et.al, in his study of monocular surgery for large angle strabismus observed the range of pre-operative deviation in exotropia patients to be 40 to 80 PD with the mean pre-operative deviation of 54.79 ± 11.15 PD. All patients with pre-operative deviation of up to 60 PD underwent successful surgeries (postoperative deviation of ≤15 PD). Some patients with pre-operative deviations of 65 PD and all patients with the deviations over 65 PD had residual deviations over 15 PD. So according to him monocular surgery can be an alternative for horizontal large-angle strabismus given deviations of up to 60 PD, however it did not result in successful outcomes for deviations of over 65 PD [30]. Gezer in his study analyzed the patient’s data to determine the factors influencing surgical success. He found pre-operative deviation being the most important factor in determining favorable outcome in patients whose exotropia was treated with surgery.

Accordingly, patients with larger pre-operative deviations had a poorer chance of successful outcome after a single surgical intervention [19].

In a study performed by Kushner in an attempt to define the factors influencing patient response to strabismus surgery, it was shown pre-operative deviation was the most important determining factor [31].

Most investigators accept the value of the pre-operative deviation as an important determinant of postoperative alignment/successful outcome. But there is no specific cutoff of pre-operative deviation to achieve surgical success with the two muscles, as it also depends on some other factors such as the surgical dose, surgical technique and it also varies a little from surgeon to surgeon. In our study, patients with pre-operative deviations >70 PD had less favorable outcomes. We believe that such patients require more than two muscles to be operated upon in order to achieve more favorable results.

## Conclusion

We found pre-operative deviation to be the strongest predictor for favorable outcome. Therefore, eliminating the factors causing error in the correct determination of pre-operative deviation should improve the success and predictability of the surgical outcome. Despite the obstacles in the surgical management of strabismus, our results are encouraging. In effect, we obtained post operative alignment or success of ≤10 PD in pre-operative deviations up to 70 PD with two muscle surgeries. The patients thus get significant aesthetic improvement. However deviations >70 PD can be more reliably served with a 3 or 4-muscle procedure. Limitations of this study are the lack of a comparative group and that we followed the patients for only six months post operatively. Thus future analysis can be done of patients with preoperative deviations above 70 PD treated with 3-muscle procedures and with longer post operative follow-up to determine a long term cure.

## Competing interests

The authors declare that they have no competing interests.

## Authors’ contributions

QAS did the assessment and surgeries, conceived the study, conducted the Literature search, designed the study and formulated the Performa, collected the Data, converted The data in SPSS file and drafted the main manuscript. AD and MAT assisted in overall study. AC assisted in surgeries and overall management of the patients. TMK also did Assessment of the patients pre and post operatively. JN served as a Supervisor and kept track of the timelines. All authors read and approved the final manuscript.
